# Temporal trend of vasectomies in Brazil and its regions by age group and race/skin color: a temporal analysis from 2013 to 2022

**DOI:** 10.1590/S2237-96222024v34e20240209.en

**Published:** 2025-05-12

**Authors:** Alessandro Vidal de Oliveira, Ana Luiza Nepomuceno Sampaio, Rui Wanderley Mascarenhas

**Affiliations:** 1 Universidade do Estado do Pará, Faculdade de Medicina, Belém, PA, Brazil; 2 Universidade do Estado do Pará, Departamento de Saúde Integrada, Belém, PA, Brazil

**Keywords:** Vasectomy, Sterilization, Reproductive, Contraception, Time Series Studies, Health Information Systems, Vasectomía, Esterilización Reproductiva, Anticoncepción, Estudios de Series Temporales, Sistemas de Información en Salud

## Abstract

**Objective:**

To analyze the temporal trend of vasectomies in Brazil and its regions by age group and race/skin color from 2013 to 2022.

**Methods:**

This is a time series analysis study based on Hospital Information System data. Average annual percentage change (AAPC) and 95% confidence intervals (95%CI) of vasectomy coefficients were estimated using Joinpoint regression. Trends were compared using parallel testing and coincidence testing.

**Results:**

In the period, there were 309,047 vasectomies in Brazil and their trend was stationary (AAPC 5.57; 95%CI -1.08; 12.66), although there was a rising trend in the Northern region (AAPC 11.53; 95%CI 2.30; 21.59) and in the Northeast region (AAPC 8.90; 95%CI 1.94; 16.34). All races/skin colors showed rising trends. Men who were 50-54 years old (AAPC 8.69; 95%CI 1.14; 16.81) and 55-59 years old (AAPC 8.71; 95%CI 0.92; 17.10) had the highest AAPC as well as rising trends. There were differences in trends, especially between age groups.

**Conclusion:**

Vasectomy trends varied across Brazil, highlighting regional, age and racial disparities related to this procedure.

## Resumen

**Objetivo:**

Analizar la tendencia temporal de las vasectomías en Brasil y regiones por grupo de edad y raza/color de piel de 2013 a 2022.

**Métodos:**

Estudio de análisis de series temporales con datos del Sistema de Información Hospitalaria. El cambio porcentual anual promedio (CPAP) y el intervalo de confianza del 95% (IC95%) de los coeficientes de vasectomía se estimaron mediante la regresión de Joinpoint. Las tendencias se compararon mediante pruebas de paralelismo y coincidencia.

**Resultados:**

Hubo 309.047 vasectomías en Brasil cuya tendencia fue estacionaria (CPAP 5,57; IC95% -1,08; 12,66), sin embargo, aumentó en el Norte (CPAP 11,53; IC95% 2,30; 21,59) y en el Nordeste (CPAP 8,90; IC95% 1,94; 16,34). Todas las razas y colores de piel mostraron tendencias crecientes. Las poblaciones de 50 a 54 años (CPAP 8,69; IC95% 1,14; 16,81) y de 55 a 59 años (CPAP 8,71; IC95% 0,92; 17,10) tuvieron las CPAP más altas y tendencias ascendentes. Hubo diferencias en las tendencias, especialmente entre grupos de edad.

**Conclusión:**

Las tendencias de la vasectomía variaron en todo Brasil, destacando las disparidades regionales, etarias y raciales relacionadas con el procedimiento.

## Introduction

Family planning depends directly on adequate guidance on contraceptive methods, sexually transmitted infections and unwanted pregnancies (1), however, more than 50% of pregnancies are unplanned in Brazil (2). Despite the reduced number of contraceptive methods available for the male population, which are limited to barrier and surgical methods, surgical sterilization is one of the safest, simplest and most effective means of preventing conception (3). Furthermore, this procedure has an excellent cost-benefit ratio when compared to other methods (3,4).

Vasectomy consists of making an incision in the scrotum in order to cut and remove a section of the vas deferens (4), which is a simple procedure. Despite the existence of social stigma linked to its being performed, sterilization has had relative acceptance and interest among the male population (3). However, individuals who have undergone vasectomy often report the need to overcome fear and judgment from friends and family when deciding to undergo sterilization (5). Men who would benefit most from vasectomies are between 15 and 60 years old, since aging modifies the quality, morphology and concentration of sperm (6,7), reducing cumulative pregnancy rates (7) and the amount of sperm in the seminiferous tubules after 50 years of age (8).

Despite its importance, there is a limited number of studies that discuss this topic in Brazil, even though male sterilization has one of the lowest failure rates and the highest cost-benefit ratio (4,9). This method is responsible for saving almost USD 14,000 by preventing unwanted pregnancies (9). Trends in vasectomies vary around the world (3); however, as far as we know, there is no literature that discusses this particular issue in Brazil. Recognizing changes in the temporal pattern of vasectomies helps to gain a better understanding of the contraception scenario in Brazil, as men currently seek fatherhood when they are older (6), however, there is inequality in the prevalence of vasectomies depending on race/skin color, and is more common among White men (3). Furthermore, the few national studies (5,10-13) that discuss this topic do not have trend analysis as their objective.

As such, the objective of this article was to analyze the temporal trend of vasectomies in Brazil and its regions by age group and race/skin color from 2013 to 2022.

## Methods

### Design and setting

This is a time series analysis study of vasectomies performed within the Brazilian National Health System (*Sistema Único de Saúde* - SUS) from 2013 to 2022, using data from the Hospital Information System (14), available via the SUS Information Technology Department (DATASUS). 

Brazil is a country located in South America, with a territorial area of ​​8,510,417.77 km², made up of 27 Federative Units, i.e. 26 states and the Federal District. The Federative Units are distributed across five regions or macro-regions characterized by geographic, human, economic and/or social similarities: North, Northeast, Southeast, South and Midwest. Currently, Brazil’s population is estimated at approximately 203 million inhabitants, 48.5% of whom are male (15).

### Participants

We analyzed the number of vasectomies performed on males between 20 and 59 years old in the SUS in the Brazilian regions. In the SUS, despite the existence of the ICD-10 code, most physicians use the standardized SUS procedure code and not the respective ICD-10 code. Therefore, we used the Hospital Information System vasectomy procedure code (04060402240) to obtain the data. All procedures performed in private practices were excluded. Additionally, unknown and incorrectly tabulated data contained in the dataset were also excluded from the analysis.

### Variables

We calculated the vasectomy coefficient per 100,000 inhabitants based on demographic data available from DATASUS and the Brazilian Institute of Geography and Statistics (16,17), dividing the annual number of procedures by the number of male residents in the regions. The following variables were analyzed:

- Age group (in years): 20-24, 25-29, 30-34, 35-39, 40-44, 45-49, 50-54, 55-59;

- Race/skin color: White, Black and mixed race;

- Federative Units of residence: Acre, Alagoas, Amapá, Amazonas, Bahia, Ceará, Distrito Federal, Espírito Santo, Goiás, Maranhão, Mato Grosso, Mato Grosso do Sul, Minas Gerais, Pará, Paraíba, Paraná, Pernambuco, Piauí, Rio de Janeiro, Rio Grande do Norte, Rio Grande do Sul, Rondônia, Roraima, Santa Catarina, São Paulo, Sergipe and Tocantins;

- Regions of residence: North, Northeast, Southeast, South and Midwest.

### Data sources

Vasectomy data were extracted from DATASUS in June 2023 from hospital production data using the microdatasus package (18), developed for R software v4.1.2. The demographic data for residents in each Federative Unit, region and age group were also extracted from DATASUS (16), while demographic data regarding race/skin color were obtained from the Brazilian Institute of Geography and Statistics (17).

### Statistical methods

We calculated annual percentage change (APC), average annual percentage change (AAPC) and 95% confidence intervals (95%CI) for the vasectomy coefficients by region of residence, Federative Unit of residence, age group and race/skin color using Joinpoint regression (19). This form of regression compares whether several straight line segments describe the data trend better when compared to a simple straight line, whereby inflection points are estimated by Monte Carlo permutation and chosen by the model with the lowest Bayesian information criterion (19). Furthermore, trends were compared using paired coincidence and parallel tests to identify the existence of disparities within the Federative Units.

The paired comparison analyzed the existence of disparities in the trends of the Federative Units due to segregation into two age groups (20-39 years vs. 40-49 years) and two races/skin colors (White versus mixed race and Black). Coincidence tests assessed whether regression models produced via Joinpoint regression were identical for a single intercept, while parallel tests assessed whether regression models were parallel but with different intercepts. Pairwise comparisons were calculated using a permutation model that estimated p-values ​​by means of a modified F-test (20).

Trends were classified as rising, when APC or AAPC were positive with a positive 95%CI; falling, when APC or AAPC were negative with a negative 95%CI; and stationary, when the 95%CI included the value zero, despite the APC or AAPC values. Paired comparisons were classified as non-coincident or non-parallel when the p-valor<0.050. For descriptive statistics, the annual frequency of each of the variables of interest was calculated together with their percentage, in addition to the average annual age of the population undergoing vasectomies together with their respective standard deviations (SD), which were reported in a table.

Trend analysis was performed using Joinpoint v4.9.1.0 software with log-linear transformation of data and 95%CI estimation using parametric methods. The year was taken as the independent variable and the vasectomy coefficient was taken as the dependent variable; in addition, 4,499 repetitions were considered in permutation tests. The model accounted for heteroskedasticity by calculating the standard error; however, it did not account for autocorrelation, as it could have reduced the power to detect inflection points (19). All other analyses were performed using R v4.1.2 with p-value<0.050 and two-tailed tests.

## Results

Overall, 309,047 vasectomies were performed in Brazil during the period analyzed. Mean age was 37.2 years (SD 6.8). Vasectomies were predominantly performed on White individuals (48.1%) and on individuals in the 35-39 age group (27.8%). A slight increase in average age from 36.5 (SD 6.7) in 2013 to 37.8 (SD 6.9) can be seen in 2022. Unknown data for the race/skin color variable was an important factor, with frequencies ranging from 22.5% to 41.6%. The remaining information about the characteristics of vasectomies is described in Table 1.

**Table 1:** Absolute frequencies (n), relative frequencies (%), average annual ages and respective standard deviations (SD) for vasectomies performed, by region of residence, race/skin color and age group. Brazil, 2013-2022

Variable	Year procedure performed									
	2013	2014	2015	2016	2017	2018	2019	2020	2021	2022
**Number of vasectomies**	23,872	25,221	25,834	26,046	30,204	37,737	41,780	21,259	25,402	51,692
**By region of residence**	**n** (%)	**n** (%)	**n** (%)	**n** (%)	**n** (%)	**n** (%)	**n** (%)	**n** (%)	**n** (%)	**n** (%)
North	458 (1.9)	559 (2.2)	685 (2.7)	688 (2.6)	1,031 (3.4)	1,363 (3.6)	831 (2.0)	707 (3.3)	923 (3.6)	2,106 (4.1)
Northeast	3,071 (12.9)	4,412 (17.5)	4,351 (16.8)	4,516 (17.3)	4,606 (15.2)	5,829 (15.4)	7,140 (17.1)	3,837 (18.0)	5,125 (20.2)	10,343 (20.0)
Southeast	13,857 (58.0)	14,125 (56.0)	14,592 (56.5)	14,449 (55.5)	16,016 (53.1)	21,805 (57.8)	22,956 (54.9)	11,942 (56.2)	13,588 (53.5)	26,382 (51.0)
South	5,040 (21.1)	4,661 (18.5)	4,546 (17.6)	4,275 (16.4)	6,164 (20.4)	6,343 (16.8)	7,835 (18.8)	3,475 (16.3)	4,100 (16.1)	9,916 (19.2)
Midwest	1,446 (6.1)	1,464 (5.8)	1,660 (6.4)	2,118 (8.1)	2,387 (7.9)	2,397 (6.4)	3,018 (7.2)	1,298 (6.2)	1,666 (6.6)	2,945 (5.7)
**By race/skin color** ^a,b^										
White	7,898 (33.1)	7,922 (31.4)	7,768 (30.1)	7,800 (29.9)	10,291 (34.1)	11,941 (31.6)	14,388 (34.4)	7,274 (34.2)	8,187 (32.2)	17,590 (34.0)
Black	840 (3.5)	752 (3.0)	853 (3.3)	893 (3.4)	1,089 (3.6)	1,581 (4.2)	1,768 (4.2)	1,143 (5.4)	1,143 (4.5)	2,098 (4.1)
Mixed race	5,246 (22.0)	6,312 (25.0)	6,211 (24.0)	7,121 (27.3)	8,713 (28.8)	10,841 (28.7)	12,210 (29.2)	6,598 (31.0)	8,414 (33.1)	19,802 (38.3)
Unknown	9,795 (41.0)	10,137 (40.2)	10,751 (41.6)	9,592 (36.8)	9,383 (31.1)	12,439 (33.0)	12,461 (29.8)	5,514 (25.9)	7,108 (28.0)	11,614 (22.5)
**By age group** (years)										
20-24	162 (0.7)	189 (0.7)	164 (0.6)	180 (0.7)	159 (0.5)	214 (0.6)	227 (0.5)	129 (0.6)	146 (0.6)	314 (0.6)
25-29	3,445 (14.4)	3,503 (13.9)	3,531 (13.7)	3,373 (13.0)	3,648 (12.1)	4,559 (12.1)	4,776 (11.4)	2,392 (11.3)	2,866 (11.3)	5,710 (11.0)
30-34	6,578 (27.6)	6,989 (27.7)	7,031 (27.2)	6,855 (26.3)	7,592 (25.1)	9,465 (25.1)	10,115 (24.2)	5,083 (23.9)	6,057 (23.8)	11,604 (22.4)
35-39	6,399 (26.8)	6,717 (26.6)	6,969 (27.0)	7,250 (27.8)	8,570 (28.4)	10,696 (28.3)	11,872 (28.4)	6,021 (28.3)	7,287 (28.7)	14,170 (27.4)
40-44	4,297 (18.0)	4,489 (17.8)	4,774 (18.5)	4,807 (18.5)	5,793 (19.2)	7,349 (19.5)	8,543 (20.4)	4,451 (20.9)	5,206 (20.5)	11,225 (21.7)
45-49	1,997 (8.4)	2,266 (9.0)	2,266 (8.8)	2,405 (9.2)	2,915 (9.7)	3,629 (9.6)	4,220 (10.1)	2,094 (9.8)	2,539 (10.0)	5,669 (11.0)
50-54	727 (3.0)	839 (3.3)	813 (3.1)	897 (3.4)	1,157 (3.8)	1,401 (3.7)	1,531 (3.7)	821 (3.9)	970 (3.8)	2,214 (4.3)
55-59	267 (1.1)	229 (0.9)	286 (1.1)	279 (1.1)	370 (1.2)	424 (1.1)	496 (1.2)	268 (1.3)	331 (1.3)	786 (1.5)
**Average age** (years) SD	36.5 (6.7)	36.5 (6.7)	36.7 (6.7)	36.9 (6.7)	37.2 (6.8)	37.1 (6.7)	37.4 (6.7)	37.4 (6.8)	37.4 (6.7)	37,8 (6.9)

^a^The percentage (%) of the category is not equal to 100% because Asian and Indigenous race/skin color was not included in the analysis due to lack of data; ^b^There were 98,794 vasectomies for which race/skin color was unknown.

**Figure 1:**
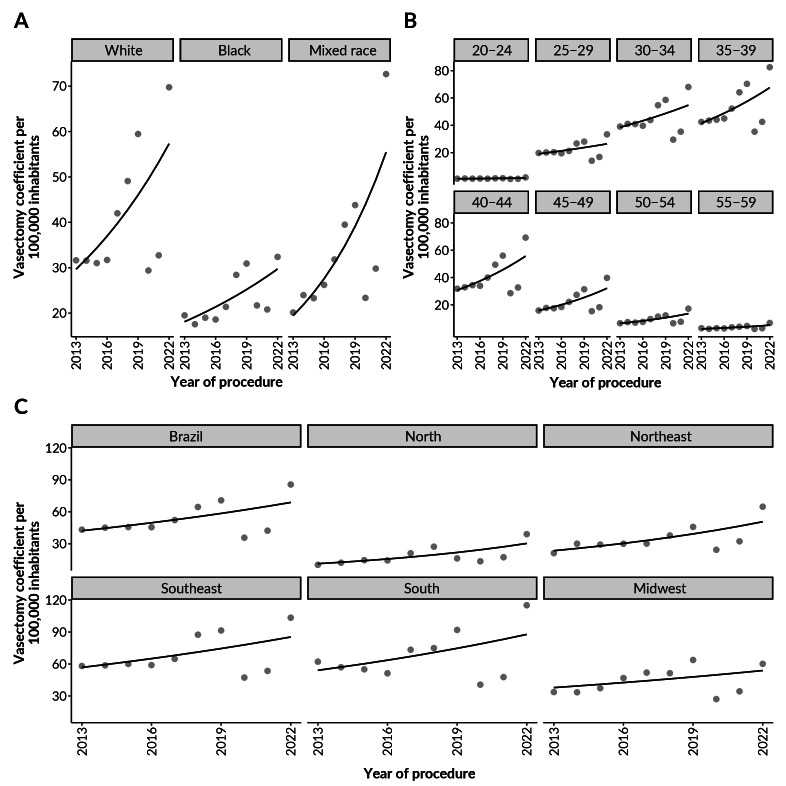
Time series of observed and modeled values represented by dots and lines, respectively, for vasectomy coefficient per 100,000 inhabitants, according to race/skin color (A), age group in years (B), and region of residence (C). Brazil, 2013–2022

Figure 1 illustrates the trends observed in the period analyzed in which the points represent the real values ​​of the vasectomy coefficient, while the lines represent the modeled values, in which there is an absence of inflection points in all the series analyzed.

The highest vasectomy coefficients were observed among males of White and mixed race/skin color (Figure 1A), with both coefficients reaching their highest values ​​in 2022 (Whites: 69.7 per 100,000 inhabitants; mixed race: 72.6 per 100,000 inhabitants). Although males of Black race/skin color had a lower vasectomy coefficient compared to other races/skin colors, they also reached their highest coefficient in 2022 (33.4 per 100,000 inhabitants). Across all age groups (Figure 1B), the highest vasectomy coefficients were observed in the 35-39 age group, and its highest coefficient occurred in 2022 (82.6 per 100,000 inhabitants), while the lowest occurred in 2020 (35.4 per 100,000 inhabitants). However, we found that all age groups had their lowest values ​​in 2020.

Brazil as a whole had a vasectomy coefficient of 43.2 per 100,000 inhabitants in 2013 and 85.6 per 100,000 inhabitants in 2022, with the lowest coefficient recorded in 2019 (63.7 per 100,000 inhabitants). Furthermore, the country’s Southeast and Southern regions had the highest coefficients among all five Brazilian regions (Figure 1C), with higher coefficients in the Southern region in 2022 (115.1 per 100,000 inhabitants). Only the Midwest region had its highest vasectomy coefficient in 2019 (63.7 per 100,000 inhabitants), while all other regions had their highest coefficients in 2022.

The vasectomy coefficient trend was stationary for Brazil as a whole (AAPC 5.57; 95%CI -1.08; 12.66), however, the Northern region (AAPC 11.53; 95%CI 2.30; 21.59) and the Northeast region (AAPC 8.90; 95%CI1.94; 16.34) had rising trends. All races/skin colors had rising trends, with greater variation among mixed race males (AAPC 12.34; 95%CI 3.99; 21.35). With regard to age groups, only the 45-49 (AAPC 8.17; 95%CI 0.71; 16.18), 50-54 (AAPC 8.69; 95%CI 1.14; 16.81) and 55-59 age groups (AAPC 8.71, 95%CI 0.92; 17.10) had rising trends. The remaining variations and their trends are described in Table 2.

**Table 2:** Trends, average annual percentage changes and 95% confidence intervals (95%CI) of vasectomy coefficients per 100,000 inhabitants, by region of residence, race/skin color and age group. Brazil, 2013-2022

Variable	Change	95%CI	p-value	Trend
**Region of residence**				
Brazil	5.57	-1.08; 12.66	0.091	Stationary
North	11.53	2.30; 21.59	0.019	Rising
Northeast	8.90	1.94; 16.34	0.018	Rising
Southeast	4.63	-1.82; 11.51	0.140	Stationary
South	5.53	-2.27; 13.94	0.145	Stationary
Midwest	4.00	-3.26; 11.82	0.247	Stationary
**Race/skin color**				
White	7.59	0.34; 15.35	0.042	Rising
Black	5.65	0.74; 10.81	0.029	Rising
Mixed race	12.34	3.99; 21.35	0.008	Rising
**Age group** (years)				
20-24	4.67	-1.90; 11.68	0.143	Stationary
25-29	3.65	-2.55; 10.25	0.217	Stationary
30-34	3.96	-2.22; 10.54	0.182	Stationary
35-39	5.54	-1.07; 12.60	0.091	Stationary
40-44	6.82	-0.24; 14.38	0.057	Stationary
45-49	8.17	0.71; 16.18	0.035	Rising
50-54	8.69	1.14; 16.81	0.028	Rising
55-59	8.71	0.92; 17.10	0.032	Rising

**Figure 2:**
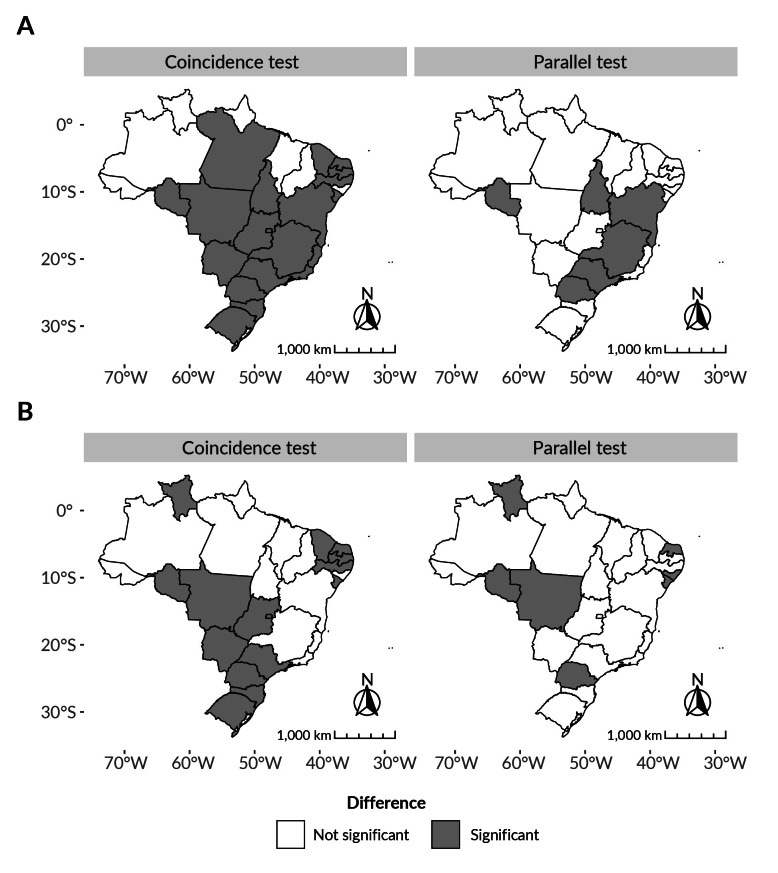
Pairwise comparison of vasectomy coefficient trends per 100,000 inhabitants across age groups (A) and race/skin color (B) categories, based on coincidence and parallel tests and federative units. Brazil, 2013–2022

Non-parallel trends (Figure 2A) were observed especially in the Federative Units located in Southeast Brazil when compared to age groups. Among the Federative Units with non-parallel trends, the highest AAPCs were observed in the 40-59 age group, with Rondônia (AAPC 17.94; 95%CI 4.55; 33.03) and Bahia (AAPC 17.04; 95%CI 7.12; 27.88) showing the largest variations. Only Alagoas, Bahia, Ceará, Espírito Santo, Mato Grosso, Pará and Santa Catarina showed rising trends. In addition, only Acre (AAPC 7.67; 95%CI -15.48; 37.16), Maranhão (AAPC 36.53; 95%CI 19.08; 56.54), Pará (AAPC 23.31; 95%CI 15.40; 31.76), Piauí (AAPC 24.43; 95%CI 0.94; 53.39) and Roraima (AAPC 8.06; 95%CI 0.76; 15.89) had higher AAPC for the 20-39 age groups.

Furthermore, a cluster of Federative Units with coinciding trends can be seen in areas in Northern Brazil, so that there is a combined trend between the two age groups (Figure 2A). Acre (AAPC 4.95; 95%CI -9.07; 21.14), Roraima (AAPC 4.56; 95%CI -0.48; 9.85) and Amazonas (AAPC 17.53; 95%CI -3.24; 42.77) showed stationary trends, with the latter having an inflection point in 2016, with mitigation of its initial trend (APC 58.19; 95%CI -14.11; 191.35) in relation to its final trend (APC 1.31; 95%CI -7.72; 11.23).

Non-parallel trends between races/skin colors were more dispersed across the Brazilian territory (Figure 2B) than in the analysis by age group. The Federative Units that showed rising trends only among males of Black and mixed race/skin color were Alagoas (AAPC 64.02; 95%CI 32.89; 102.44), Mato Grosso (AAPC 10.15; 95%CI 3.01; 17.79), Paraná (AAPC 25.03; 95%CI 10.64; 41.29) and Sergipe (AAPC 13.00; 95%CI 1.37; 25.98). However, Rondônia showed a rising trend only among males of White race/skin color (AAPC 28.55; 95%CI 0.07; 65.14), while Rio Grande do Norte showed stationary trends for both racial groups, with greater variation among White males (AAPC 10.58; 95%CI -12.71; 40.08). It was not possible to assess trends among White males in Roraima, as no vasectomies were performed for them.

A range of coinciding trends can be seen in the Brazilian territory from the North to the Southeast, making it possible to build a combined trend for the two groups analyzed (Figure 2B). Among the Federative Units with coinciding trends, those with the highest AAPC with rising trends were Alagoas (AAPC 56.02; 95%CI 35.11; 80.17), Maranhão (AAPC 50.70; 95%CI 34.69; 68.61), Amapá (AAPC 34.07; 95%CI 12.51; 59.76) and Pará (AAPC 25.21; 95%CI 18.43; 32.39). The combined trends in Acre (AAPC 10.93; 95%CI -14.09; 43.25), Minas Gerais (AAPC 9.81; 95%CI -0.25; 20.89) and Rio de Janeiro (AAPC 11.47; 95%CI -3.48; 28.73) were stationary. As with the parallel test, it was not possible to analyze Roraima in the coincidence test.

## Discussion

We analyzed vasectomy coefficient trends in Brazil by age group and race/skin color from 2013 to 2022. The vasectomy coefficient trend per 100,000 inhabitants was stationary in Brazil as a whole. The highest vasectomy coefficient was found in the Southern region of the country, among mixed race males and those in the 35-39 age group. The trends were rising among all races/skin colors, however, they were variable when age groups were analyzed. There were disparities in trends between the Federative Units analyzed when comparing race/skin color and age groups.

The aggregate trend for Brazil as a whole was stationary. However, the vasectomy coefficient trends varied in the country’s regions, corresponding to the reality found in other countries (3). In the United Kingdom (21), the vasectomy coefficient decreased by 62.3% from 2004 to 2022, while there were inconsistent trends in the United States (22,23). These inconsistencies are also noticeable between Brazilian regions, with higher vasectomy coefficients in the Southern and Southeast regions; although with stationary trends in these regions and rising trends in the North and Northeast regions.

The heterogeneity of Brazilian regions and Federative Units needs to be taken into account, given that social, economic and cultural differences are related to changes in reproductive health (24). In addition to these factors, changes in laws and changes in gender roles influence the decision-making process regarding reproductive rights, such as the change in the law that regulates sterilization in Brazil in 2022 (25) and the overturning of the constitutional right to abortion in the United States in the same year, where there was a greater demand for vasectomy procedures, especially by childless and younger individuals (26).

The influence of court decisions can be positive or negative to the extent that the change in the reproductive rights of given a population allows the other spectrum to adapt, indirectly reflecting on health care decisions (26). However, the existence of regulatory laws does not guarantee full access by the target population, as there are illegal variations in the criteria adopted by some municipal governments for performing sterilization (13), making the process more difficult for people who are unaware of their rights as guaranteed by law.

There were higher numbers of vasectomies among Brazilian males aged between 30 and 44, with a progressive annual increase, especially among those over 35 years of age. Most sterilizations are performed among those in the 30-49 age group (11,21,27,28), among whom the main factors associated with the decision to have a vasectomy are linked to lack of confidence in other contraceptive methods, being certain of their inability to get their partner pregnant (5), ease of carrying out the procedure and not wishing to have more offspring (11). However, the occurrence of a lower annual number of vasectomies, lower vasectomy coefficients and lower AAPC among Black males makes the hypothesis of continuing inequities in access to health and information in this specific population plausible.

Stationary trends were found among younger males (20-44 years old) and rising trends among males over 45 years old. The progressive increase in paternal age among the male population (6) is linked to greater time dedicated to formal education (24), which is reflected in the slight increase in the average age of males who underwent vasectomies in Brazil and in the rising trends in vasectomy rates, especially among individuals over 45 years old, as the literature indicates that the male population is tending to procreate at a progressively older age than in the past (6).

Differences were found between trends in the Federative Units we analyzed, both when comparing age groups and when comparing race/skin color. These disparities can be partially explained by the greater absolute number of vasectomy procedures undergone by people with a higher level of education and by those living in urban areas in Latin American countries (3), in addition to positive association between the absolute number of procedures and per capita income (29). This would therefore explain the greater absolute number of procedures undergone by people who lived in the Southeast region of Brazil, since aspects linked to economic and social development permit greater access to different contraceptive methods (24).

However, the relationship between the difference between urban and rural regions in terms of vasectomy trends is not very explicit in the literature. Two studies carried out in the United States from 2014-2021 (23) and 2011-2017 (30) found differences between urban and rural regions, with a greater relative increase among those residing in rural regions (34%) (23), lower average age of those having vasectomies and a greater number of children (30); while another study, also conducted in the United States in 2007-2013 (27), found no difference between the vasectomy coefficients of urban and rural regions. These diverging findings can be attributed to the different analysis periods, which may be associated with legislative, behavioral, socioeconomic and cultural changes in the society studied, highlighting the multiple variables that influence the decision-making process regarding choice of the ideal contraceptive method (24). Urbanization, in turn, appears to exert a double influence on trends, as it increases the supply of health services in urban areas while increasing discrepancies between urban and rural regions (24).

Moreover, there was a considerable proportion of unknown data for the race/skin color variable in the period studied. Despite the apparent reduction in the proportion of unknown data from 2013 to 2022, this variable was not adequately filled in on the database in any of the years analyzed, according to the classification made by Romero & Cunha (31). Similarly to our findings, a national study covering the period 2010-2012 (32) and another study covering the period 2009-2018 (33) found 35% and 29% incompleteness for this variable, respectively. The continuing incompleteness of this indicator may therefore be linked to the fact that production of information in Brazil is unorganized and unsystematic, especially in the North, Northeast and Midwest regions, possibly introducing biases in analyses, as well as distorting both information and reality (33).

This study is not without limitations, the main ones being related to its design, which made analysis at the individual level impossible. Furthermore, the unavailability of demographic data on other races/skin colors prevented other populations from being included. The data source may be a limitation due to incorrect data input and vasectomy procedure underreporting, especially given the amount of unknown data for the race/skin color variable, which could have provided important information about the living and health conditions of those for whom this data was recorded as unknown (32), which may have led to shortcomings in interpreting the results. Finally, analyzing countries like Brazil, which has a large territory and a high degree of heterogeneity among its population, may be a limitation, since places – especially in the North and Northeast regions of the country – with a smaller population size and greater difficulty in accessing health care may present greater underreporting (34). However, the analysis was divided into regions, Federative Units, age groups and race/skin color with the aim of minimizing this limitation.

Despite its limitations, this study is the first to quantify vasectomy trends in Brazil and its regions, being essential for understanding the contraception scenario in Brazil, especially after the change in the 2022 law that regulates sterilizations in Brazil (25). It will, therefore, enable future studies to use the data presented in this manuscript as an object of comparison between two distinct periods: before and after the change in the law. The law in question changes the minimum age for requesting sterilization in Brazil from 25 to 21 years (25), which may influence the population’s decision-making process, especially among younger generations. Finally, the APC, AAPC and their trends provided in this study are essential for evaluating factors related to changes in patterns of increase or decrease in the number of vasectomies.

To conclude, the rising trends observed may be linked to changes in reproductive decisions, possibly being influenced by greater awareness about family planning, greater access to specialized health services, greater reliability of the method, in addition to the reduction of stigma associated with vasectomy. However, regional, age and racial disparities highlight the importance of targeted public policies and specific strategies for each population, aiming to promote equity in access to reproductive health services, as well as encouraging family planning throughout Brazil.

## Data Availability

The data used in this study is available in full at https://datasus.saude.gov.br/.
