# Supplementary material for: Temporal trend of vasectomies in Brazil and its regions by age group and race/skin color: a temporal analysis from 2013 to 2022
Source: Epidemiol Serv Saude. 2025 May 12;34:e20240209. doi: 10.1590/S2237-96222024v34e20240209.en (PMC12077542; doi:10.1590/S2237-96222024v34e20240209.en)
Supplement: Translation

# Translation: Tendência temporal de vasectomias no Brasil e regiões por faixa etária e raça/cor da pele: uma análise temporal de 2013 a 2022

**DOI:** 10.1590/S2237-96222024v34e20240209.pt

**Authors:** Alessandro Vidal de Oliveira, Ana Luiza Nepomuceno Sampaio, Rui Wanderley Mascarenhas

**Affiliations:** 1 Universidade do Estado do Pará, Faculdade de Medicina, Belém, PA, Brasil; 2 Universidade do Estado do Pará, Departamento de Saúde Integrada, Belém, PA, Brasil

**Keywords:** Vasectomia, Esterilização reprodutiva, Anticoncepção, Estudos de séries temporais, Sistemas de informação em saúde

## Abstract

**Objetivo:**

Analisar a tendência temporal das vasectomias no Brasil e regiões por faixa etária e raça/cor da pele de 2013 a 2022.

**Métodos:**

Estudo de análise de séries temporais com dados do Sistema de Informações Hospitalares. Estimou-se a variação percentual anual média (VPAM) e o intervalo de confiança de 95% (IC95%) dos coeficientes de vasectomia por regressão Joinpoint. Comparou-se as tendências pelos testes de paralelismo e de coincidência.

**Resultados:**

Houve 309.047 vasectomias no Brasil cuja tendência foi estacionária (VPAM 5,57; IC95% -1,08; 12,66), porém, crescente no Norte (VPAM 11,53; IC95% 2,30; 21,59) e Nordeste (VPAM 8,90; IC95% 1,94; 16,34). Todas as raças/cores da pele apresentaram tendências crescentes. As populações de 50-54 (VPAM 8,69; IC95% 1,14; 16,81) e 55-59 anos (VPAM 8,71; IC95% 0,92; 17,10) apresentaram as maiores VPAM e tendências crescentes. Houve diferença nas tendências, sobretudo entre as faixas etárias.

**Conclusão:**

As tendências de vasectomias variaram no território brasileiro, evidenciando disparidades regionais, etárias e raciais relacionadas ao procedimento.

## Introdução

O planejamento reprodutivo depende diretamente da orientação adequada sobre métodos contraceptivos, infecções sexualmente transmissíveis e gravidezes indesejadas (1), contudo, mais de 50% das gravidezes não são planejadas no Brasil (2). Apesar do número reduzido de métodos contraceptivos disponíveis para a população masculina, que se limitam a métodos de barreira e cirúrgicos, a esterilização cirúrgica é um dos meios mais seguros, simples e eficazes para prevenir a concepção (3). Além disso, este procedimento apresenta uma ótima relação custo-benefício quando comparado a outros métodos (3,4).

A vasectomia consiste na secção dos ductos deferentes por meio de uma incisão escrotal (4), refletindo na simplicidade do procedimento. Apesar da existência de estigma social atrelado a sua realização, a esterilização apresenta relativa aceitação e interesse pela população masculina (3). No entanto, indivíduos que se submeteram à vasectomia frequentemente relatam a necessidade de superar o medo e o julgamento por parte de amigos e familiares ao decidir pela esterilização (5). A população que mais se beneficiaria das vasectomias tem entre 15 e 60 anos, pois o envelhecimento modifica a qualidade, morfologia e concentração dos espermatozoides (6,7), reduzindo as taxas cumulativas de gravidez (7) e o número de espermatozoides nos túbulos seminíferos após os 50 anos de idade (8).

Apesar de sua importância, há um número limitado de estudos que discutem este tópico no Brasil, mesmo que a esterilização masculina apresente uma das menores taxas de falha e a maior relação custo-benefício (4,9). Este método é responsável por uma economia de quase 14.000 dólares ao prevenir gravidezes indesejadas (9). As tendências em vasectomias variam ao redor do mundo (3); no entanto, até onde se tem conhecimento, não há literatura que discuta essa particularidade no Brasil. O reconhecimento das alterações no padrão temporal das vasectomias ajuda a compreender melhor o cenário da anticoncepção no Brasil, visto que a população masculina atualmente busca a paternidade em idades mais avançadas (6), porém, há desigualdade na prevalência de vasectomias conforme as raças/cores da pele, com maior utilização por brancos (3). Além disso, os poucos estudos nacionais (5,10-13) que discutem este tema não incluem a análise de tendências como seu objetivo.

Portanto, este artigo teve como objetivo analisar a tendência temporal das vasectomias no Brasil e regiões por faixa etária e raça/cor da pele de 2013 a 2022.

## Métodos

### Desenho e contexto do estudo

Trata-se de um estudo de análise de série temporal das vasectomias realizadas no Sistema Único de Saúde (SUS) do Brasil de 2013 a 2022, utilizando dados do Sistema de Informações Hospitalares (14), disponíveis no Departamento de Informação e Informática do SUS (DATASUS). 

O Brasil é um país localizado na América do Sul, com uma área territorial de 8.510.417,77 km², composto por 27 Unidades Federativas (UF), sendo 26 estados e um distrito federal. As UF estão distribuídas em cinco regiões ou macrorregiões caracterizadas por semelhanças geográficas, humanas, econômicas e/ou sociais: Norte, Nordeste, Sudeste, Sul e Centro-Oeste. Atualmente, a população do Brasil é estimada em aproximadamente 203 milhões de habitantes, dos quais 48,5% são homens (15).

### Participantes do estudo

Analisou-se o número de vasectomias realizadas em pacientes com idades entre 20 e 59 anos no SUS nas regiões brasileiras. No SUS, apesar da existência do código CID-10, a maioria dos médicos utiliza o código padronizado do procedimento no sistema do SUS e não o respectivo código CID-10. Assim, utilizou-se o código do procedimento de vasectomia no Sistema de Informações Hospitalares (04060402240) para obter os dados. Todos os procedimentos realizados em consultórios particulares foram excluídos. Além disso, dados ignorados e incorretamente tabulados no conjunto de dados também foram excluídos da análise.

### Variáveis do estudo

Calculou-se o coeficiente de vasectomia (CVA) por 100.000 habitantes a partir dos dados demográficos disponíveis no DATASUS e no Instituto Brasileiro de Geografia e Estatística (16,17), dividindo o número anual de procedimentos pelo número de residentes do sexo masculino nas regiões. Analisou-se as seguintes variáveis:

Faixa etária (anos): 20-24, 25-29, 30-34, 35-39, 40-44, 45-49, 50-54, 55-59;

Raça/cor da pele: branca, preta e parda;

UF de residência: Acre, Alagoas, Amapá, Amazonas, Bahia, Ceará, Distrito Federal, Espírito Santo, Goiás, Maranhão, Mato Grosso, Mato Grosso do Sul, Minas Gerais, Pará, Paraíba, Paraná, Pernambuco, Piauí, Rio de Janeiro, Rio Grande do Norte, Rio Grande do Sul, Rondônia, Roraima, Santa Catarina, São Paulo, Sergipe e Tocantins;

Regiões de residência: Norte, Nordeste, Sudeste, Sul e Centro-Oeste.

### Fonte dos dados

Os dados de vasectomias foram extraídos do DATASUS em junho de 2023 a partir dos dados de produção hospitalar utilizando o pacote microdatasus (18), desenvolvido para o software R v4.1.2. Os dados demográficos dos residentes em cada UF, região e faixa etária também foram extraídos do DATASUS (16), enquanto os dados demográficos referentes à raça/cor da pele foram coletados do Instituto Brasileiro de Geografia e Estatística (17).

### Análise estatística

Calculou-se a variação percentual anual (VPA), a variação percentual anual média (VPAM) e o intervalo de confiança de 95% (IC95%) dos CVA por região de residência, UF de residência, faixa etária e raça/cor da pele utilizando a regressão Joinpoint (19). Esta regressão compara se vários segmentos de reta descrevem melhor a tendência dos dados em comparação a uma reta simples, onde os pontos de inflexão são estimados por permutação de Monte Carlo e escolhidos pelo modelo com o menor critério de informação bayesiano (19). Além disso, as tendências foram comparadas por testes pareados de coincidência e de paralelismo para identificar a existência de disparidades dentro das UF.

A comparação pareada analisou a existência de disparidades nas tendências das UF pela segregação em dois grupos etários (20-39 anos versus 40-49 anos) e duas raças/cores da pele (branca versus parda e preta). Testes de coincidência avaliaram se os modelos de regressão produzidos via regressão Joinpoint eram idênticos a um único intercepto, enquanto testes de paralelismo avaliaram se os modelos de regressão eram paralelos, mas com interceptos diferentes. As comparações pareadas foram calculadas por um modelo de permutação que estimou os p-valores por um teste F modificado (20).

As tendências foram classificadas como crescentes, quando VPA ou VPAM eram positivos com IC95% positivo; decrescentes, quando VPA ou VPAM eram negativos com IC95% negativo; e estacionárias, quando o IC95% incluía o valor zero, apesar dos valores de VPA ou VPAM. As comparações pareadas foram classificadas como não coincidentes ou não paralelas quando p-valor<0,050. Para a estatística descritiva, calculou-se a frequência anual de cada uma das variáveis de interesse em conjunto com a sua porcentagem, além da idade média anual da população submetida às vasectomias em conjunto com seus respectivos desvios-padrões (DP), os quais foram reportados em um tabela.

A análise de tendência foi realizada no software Joinpoint v4.9.1.0 com transformação log-linear dos dados e estimativa de IC95% por métodos paramétricos. Considerou-se o ano como a variável independente e o CVA como a variável dependente; além disso, considerou-se 4.499 repetições em testes de permutação. O modelo contabilizou heterocedasticidade por cálculo do erro padrão; no entanto, não contabilizou autocorrelação, pois poderia reduzir o poder de detecção dos pontos de inflexão (19). Todas as outras análises foram realizadas no software R v4.1.2 com p-valor<0,050 e testes bicaudais.

## Resultados

Foram realizadas 309.047 vasectomias no Brasil no período analisado, com uma idade média de 37,2 anos (DP 6,8). As vasectomias foram predominantemente realizadas em indivíduos brancos (48,1%) e em indivíduos na faixa etária de 35-39 anos (27,8%). É possível ver um aumento discreto da idade média de 36,5 (DP 6,7) em 2013 para 37,8 (DP 6,9) em 2022. Os dados ignorados na variável de raça/cor da pele foram um fator importante, com frequências variando de 22,5% a 41,6%. As demais informações sobre as características das vasectomias estão descritas na Tabela 1.

**Tabela 1:** Frequências absolutas (n), frequências relativas (%), idades médias anuais e seus respectivos desvios-padrões (DP) das vasectomias realizadas por região de residência, raça/cor da pele e faixa etária. Brasil, 2013-2022

Variável	Ano do procedimento									
	2013	2014	2015	2016	2017	2018	2019	2020	2021	2022
**Número de vasectomias**	23.872	25.221	25.834	26.046	30.204	37.737	41.780	21.259	25.402	51.692
**Região de residência**	**n** (%)	**n** (%)	**n** (%)	**n** (%)	**n** (%)	**n** (%)	**n** (%)	**n** (%)	**n** (%)	**n** (%)
Norte	458 (1,9)	559 (2,2)	685 (2,7)	688 (2,6)	1.031 (3,4)	1.363 (3,6)	831 (2,0)	707 (3,3)	923 (3,6)	2.106 (4,1)
Nordeste	3.071 (12,9)	4.412 (17,5)	4.351 (16,8)	4.516 (17,3)	4.606 (15,2)	5.829 (15,4)	7.140 (17,1)	3.837 (18,0)	5.125 (20,2)	10.343 (20,0)
Sudeste	13.857 (58,0)	14.125 (56,0)	14.592 (56,5)	14.449 (55,5)	16.016 (53,1)	21.805 (57,8)	22.956 (54,9)	11.942 (56,2)	13.588 (53,5)	26.382 (51,0)
Sul	5.040 (21,1)	4.661 (18,5)	4.546 (17,6)	4.275 (16,4)	6.164 (20,4)	6.343 (16,8)	7.835 (18,8)	3.475 (16,3)	4.100 (16,1)	9.916 (19,2)
Centro-Oeste	1.446 (6,1)	1.464 (5,8)	1.660 (6,4)	2.118 (8,1)	2.387 (7,9)	2.397 (6,4)	3.018 (7,2)	1.298 (6,2)	1.666 (6,6)	2.945 (5,7)
**Raça/cor d^a^ ^p^elea,b**										
Branca	7.898 (33,1)	7.922 (31,4)	7.768 (30,1)	7.800 (29,9)	10.291 (34,1)	11.941 (31,6)	14.388 (34,4)	7.274 (34,2)	8.187 (32,2)	17.590 (34,0)
Preta	840 (3,5)	752 (3,0)	853 (3,3)	893 (3,4)	1.089 (3,6)	1.581 (4,2)	1.768 (4,2)	1.143 (5,4)	1.143 (4,5)	2.098 (4,1)
Parda	5.246 (22,0)	6.312 (25,0)	6.211 (24,0)	7.121 (27,3)	8.713 (28,8)	10.841 (28,7)	12.210 (29,2)	6.598 (31,0)	8.414 (33,1)	19.802 (38,3)
Ignorada	9.795 (41,0)	10.137 (40,2)	10.751 (41,6)	9.592 (36,8)	9.383 (31,1)	12.439 (33,0)	12.461 (29,8)	5.514 (25,9)	7.108 (28,0)	11.614 (22,5)
**Faixa etária** (anos)										
20-24	162 (0,7)	189 (0,7)	164 (0,6)	180 (0,7)	159 (0,5)	214 (0,6)	227 (0,5)	129 (0,6)	146 (0,6)	314 (0,6)
25-29	3.445 (14,4)	3.503 (13,9)	3.531 (13,7)	3.373 (13,0)	3.648 (12,1)	4.559 (12,1)	4.776 (11,4)	2.392 (11,3)	2.866 (11,3)	5.710 (11,0)
30-34	6.578 (27,6)	6.989 (27,7)	7.031 (27,2)	6.855 (26,3)	7.592 (25,1)	9.465 (25,1)	10.115 (24,2)	5.083 (23,9)	6.057 (23,8)	11.604 (22,4)
35-39	6.399 (26,8)	6.717 (26,6)	6.969 (27,0)	7.250 (27,8)	8.570 (28,4)	10.696 (28,3)	11.872 (28,4)	6.021 (28,3)	7.287 (28,7)	14.170 (27,4)
40-44	4.297 (18,0)	4.489 (17,8)	4.774 (18,5)	4.807 (18,5)	5.793 (19,2)	7.349 (19,5)	8.543 (20,4)	4.451 (20,9)	5.206 (20,5)	11.225 (21,7)
45-49	1.997 (8,4)	2.266 (9,0)	2.266 (8,8)	2.405 (9,2)	2.915 (9,7)	3.629 (9,6)	4.220 (10,1)	2.094 (9,8)	2.539 (10,0)	5.669 (11,0)
50-54	727 (3,0)	839 (3,3)	813 (3,1)	897 (3,4)	1.157 (3,8)	1.401 (3,7)	1.531 (3,7)	821 (3,9)	970 (3,8)	2.214 (4,3)
55-59	267 (1,1)	229 (0,9)	286 (1,1)	279 (1,1)	370 (1,2)	424 (1,1)	496 (1,2)	268 (1,3)	331 (1,3)	786 (1,5)
**Idade média** (anos) DP	36,5 (6,7)	36,5 (6,7)	36,7 (6,7)	36,9 (6,7)	37,2 (6,8)	37,1 (6,7)	37,4 (6,7)	37,4 (6,8)	37,4 (6,7)	37,8 (6,9)

^a^A porcentagem (%) da categoria não soma 100% devido à não inclusão das raças/cores da pele amarela e indígena na análise por falta de dados; ^b^Houve 98.794 vasectomias ignoradas.

A Figura 1 ilustra as tendências observadas no período analisado em que pontos representam os valores reais do CVA e as linhas, os valores modelados, em que se observa ausência de pontos de inflexão em todas as séries analisadas.

**Figura 1:** Séries temporais dos valores observados e modelados por pontos e linhas, respectivamente, dos coeficientes de vasectomia por 100.000 habitantes, segundo a raça/cor da pele (A), faixa etária (B) e a região de residência (C). Brasil, 2013-2022

Os maiores CVA foram observados entre brancos e pardos (Figura 1A), com ambos os coeficientes atingindo os valores mais altos em 2022 (brancos: 69,7 por 100.000 habitantes; pardos: 72,6 por 100.000 habitantes). Embora a população preta tenha apresentado um CVA menor em comparação a outras raças/cores da pele, também atingiu seu maior coeficiente em 2022 (33,4 por 100.000 habitantes). Entre todas as faixas etárias (Figura 1B), os maiores CVA foram observados na faixa etária de 35-39 anos, que apresentou seu maior coeficiente em 2022 (82,6 por 100.000 habitantes) e o menor em 2020 (35,4 por 100.000 habitantes). No entanto, verificou-se que todas as faixas etárias tiveram seus valores mais baixos em 2020.

O Brasil apresentou um CVA de 43,2 por 100.000 habitantes em 2013 e de 85,6 por 100.000 hab. em 2022, com o menor coeficiente registrado em 2019 (63,7 por 100.000 habitantes). Além disso, as regiões Sudeste e Sul apresentaram os maiores coeficientes entre regiões brasileiras (Figura 1C), com coeficientes mais altos na região Sul em 2022 (115,1 por 100.000 habitantes). Apenas a região Centro-Oeste teve seu maior CVA em 2019 (63,7 por 100.000 habitantes), enquanto todas as outras regiões tiveram o maior coeficiente em 2022.

A tendência do CVA foi estacionária no Brasil (VPAM 5,57; IC95% -1,08; 12,66), no entanto, as regiões Norte (VPAM 11,53; IC95% 2,30; 21,59) e Nordeste (VPAM 8,90; IC95%1,94; 16,34) apresentaram tendências crescentes. Todas as raças/cores da pele tiveram tendências crescentes, com maior variação entre os pardos (VPAM 12,34; IC95% 3,99; 21,35). Em relação às faixas etárias, apenas as faixas etárias de 45-49 (VPAM 8,17; IC95% 0,71; 16,18), 50-54 (VPAM 8,69; IC95% 1,14; 16,81) e 55-59 anos (VPAM 8,71, IC95% 0,92; 17,10) apresentaram tendências crescentes. As demais variações e suas tendências estão descritas na Tabela 2.

**Tabela 2:** Tendências, variações percentuais anuais médias e intervalos de confiança de 95% (IC95%) dos coeficientes de vasectomia, por 100.000 habitantes, região de residência, raça/cor da pele e faixa etária. Brasil, 2013-2022

Variável	Variação	IC95%	p-valor	Tendência
**Região de residência**				
Brasil	5,57	-1,08; 12,66	0,091	Estacionária
Norte	11,53	2,30; 21,59	0,019	Crescente
Nordeste	8,90	1,94; 16,34	0,018	Crescente
Sudeste	4,63	-1,82; 11,51	0,140	Estacionária
Sul	5,53	-2,27; 13,94	0,145	Estacionária
Centro-Oeste	4,00	-3,26; 11,82	0,247	Estacionária
**Raça/cor da pele**				
Branca	7,59	0,34; 15,35	0,042	Crescente
Preta	5,65	0,74; 10,81	0,029	Crescente
Parda	12,34	3,99; 21,35	0,008	Crescente
**Faixa etária** (anos)				
20-24	4,67	-1,90; 11,68	0,143	Estacionária
25-29	3,65	-2,55; 10,25	0,217	Estacionária
30-34	3,96	-2,22; 10,54	0,182	Estacionária
35-39	5,54	-1,07; 12,60	0,091	Estacionária
40-44	6,82	-0,24; 14,38	0,057	Estacionária
45-49	8,17	0,71; 16,18	0,035	Crescente
50-54	8,69	1,14; 16,81	0,028	Crescente
55-59	8,71	0,92; 17,10	0,032	Crescente

Tendências não paralelas (Figura 2A) foram observadas especialmente nas unidades federativas localizadas no Sudeste do Brasil quando comparados às faixas etárias. Entre as UF sem tendências paralelas, as maiores VPAM foram observadas na faixa etária de 40-59 anos, sendo que Rondônia (VPAM 17,94; IC95% 4,55; 33,03) e Bahia (VPAM 17,04; IC95% 7,12; 27,88) apresentaram as maiores variações. Apenas Alagoas, Bahia, Ceará, Espírito Santo, Mato Grosso, Pará e Santa Catarina apresentaram tendências crescentes. Além disso, apenas Acre (VPAM 7,67; IC95% -15,48; 37,16), Maranhão (VPAM 36,53; IC95% 19,08; 56,54), Pará (VPAM 23,31; IC95% 15,40; 31,76), Piauí (VPAM 24,43; IC95% 0,94; 53,39) e Roraima (VPAM 8,06; IC95% 0,76; 15,89) apresentaram maiores VPAM entre as faixas etárias de 20-39 anos.

**Figura 2:** Comparação por pares das tendências do coeficiente de vasectomia por 100.000 habitantes nas faixas etárias (A) raças/cores da pele (B), conforme os testes de coincidência e de paralelismo e as Unidades Federativas, Brasil, 2013-2022

Além disso, é possível observar um aglomerado de unidades federativas com tendências coincidentes nas áreas ao norte do Brasil, permitindo a criação de uma tendência combinada entre as duas faixas etárias (Figura 2A). Acre (VPAM 4,95; IC95% -9,07; 21,14), Roraima (VPAM 4,56; IC95% -0,48; 9,85) e Amazonas (VPAM 17,53; IC95% -3,24; 42,77) apresentaram tendências estacionárias, sendo que este último teve um ponto de inflexão em 2016, com mitigação de sua tendência inicial (VPA 58,19; IC95% -14,11; 191,35) em relação a sua tendência final (VPA 1,31; IC95% -7,72; 11,23).

Tendências não paralelas entre raças/cores da pele foram mais dispersas no território brasileiro (Figura 2B) do que na análise por faixa etária. As UF que apresentaram tendências crescentes apenas entre os pardos e pretos foram Alagoas (VPAM 64,02; IC95% 32,89; 102,44), Mato Grosso (VPAM 10,15; IC95% 3,01; 17,79), Paraná (VPAM 25,03; IC95% 10,64; 41,29) e Sergipe (VPAM 13,00; IC95% 1,37; 25,98). No entanto, Rondônia apresentou tendência crescente apenas entre os brancos (VPAM 28,55; IC95% 0,07; 65,14), enquanto o Rio Grande do Norte apresentou tendências estacionárias para ambos os grupos raciais, com maior variação nos brancos (VPAM 10,58; IC95% -12,71; 40,08). Não foi possível avaliar tendências entre brancos em Roraima, pois não houve nenhum procedimento.

É possível observar uma faixa de tendências coincidentes no território brasileiro do Norte ao Sudeste, possibilitando a construção de uma tendência combinada para os dois grupos analisados (Figura 2B). Entre as UF com tendências coincidentes, as que apresentaram as maiores VPAM com tendências crescentes foram Alagoas (VPAM 56,02; IC95% 35,11; 80,17), Maranhão (VPAM 50,70; IC95% 34,69; 68,61), Amapá (VPAM 34,07; IC95% 12,51; 59,76) e Pará (VPAM 25,21; IC95% 18,43; 32,39). As tendências combinadas em Acre (VPAM 10,93; IC95% -14,09; 43,25), Minas Gerais (VPAM 9,81; IC95% -0,25; 20,89) e Rio de Janeiro (VPAM 11,47; IC95% -3,48; 28,73) foram estacionárias. Assim como no teste de paralelismo, não foi possível analisar Roraima no teste de coincidência.

## Discussão

Analisou-se as tendências dos CVA no território brasileiro por faixa etária e raça/cor da pele de 2013 a 2022. A tendência do coeficiente de vasectomia por 100.000 habitantes foi estacionária no Brasil; além disso, os maiores CVA foram observados na região Sul, entre pardos e na faixa etária de 35-39 anos. As tendências foram crescentes entre todas as raças/cores da pele, porém, foram variáveis quando se analisou as faixas etárias. Houve disparidades nas tendências entre as UF analisadas ao comparar as raças/cores da pele e as faixas etárias.

A tendência agregada do Brasil foi estacionária, porém, nas regiões brasileiras, as tendências observadas dos CVA variaram, correspondendo à realidade observada em outros países (3). No Reino Unido (21), o CVA diminuiu 62,3% de 2004 a 2022, enquanto houve tendências inconsistentes nos Estados Unidos (22,23). Essas inconsistências também são perceptíveis entre as regiões brasileiras, com maiores CVA nas regiões Sul e Sudeste; no entanto, com tendências estacionárias nessas regiões e tendências crescentes no Norte e no Nordeste.

A heterogeneidade das regiões e das UF brasileiras deve ser levada em consideração, haja vista que diferenças sociais, econômicas e culturais estão relacionadas a alterações na saúde reprodutiva (24). Além desses fatores, alterações legislativas e nos papéis de gênero influenciam o processo de tomada de decisão no que tange aos direitos reprodutivos, como a alteração da lei que regula as esterilizações no Brasil em 2022 (25) e a reversão do direito constitucional ao aborto nos Estados Unidos no mesmo ano, onde houve maior busca por procedimentos de vasectomia, principalmente por indivíduos sem filhos e mais jovens (26).

A influência das decisões judiciais pode ser positiva ou negativa à medida que a alteração dos direitos reprodutivos de uma população permite com que o outro espectro se adapte, refletindo indiretamente nas decisões de cuidados de saúde (26). No entanto, a existência de leis regulatórias não garante pleno acesso pela população alvo, já que variações ilegais nos critérios adotados pelos municípios para realizar a esterilização podem ser observadas (13), tornando o processo mais difícil para pessoas que desconhecem seus direitos garantidos por lei.

Houve maiores números de vasectomias na população brasileira entre 30 e 44 anos de idade, com progressivo aumento anual, especialmente entre a população acima de 35 anos. A principal faixa etária para a realização de esterilizações está situada entre 30 e 49 anos (11,21,27,28), em que os principais fatores associados à decisão de realizar um procedimento de vasectomia estão ligados à falta de confiança em outros métodos contraceptivos, à certeza da incapacidade de engravidar a companheira (5), à facilidade de realização do procedimento e à falta de desejo de gerar mais descendentes (11). No entanto, a ocorrência de menor número anual de vasectomias, menores CVA e menores VPAM entre pretos torna plausível a hipótese da permanência de iniquidades no acesso à saúde e à informação nesta população específica.

Encontrou-se tendências estacionárias entre populações mais jovens (20-44 anos) e tendências crescentes nas populações acima de 45 anos. O aumento progressivo da idade paterna entre a população masculina (6) está atrelado ao maior tempo dedicado à educação formal (24), o que reflete no discreto aumento da idade média dos pacientes que realizaram vasectomias no Brasil e nas tendências crescentes dos coeficientes de vasectomias, principalmente em indivíduos com mais de 45 anos, à medida que a literatura aponta que a população masculina tende a procriar em uma idade progressivamente maior do que no passado (6).

Encontrou-se diferenças entre as tendências nas UF analisadas, tanto ao comparar as faixas etárias quanto ao comparar as raças/cores da pele. Essas disparidades encontradas podem ser parcialmente explicadas pelo maior número absoluto de procedimentos realizados por pessoas com maior nível de escolaridade e por residentes de áreas urbanas em países da América Latina (3), além da associação positiva entre o número absoluto de procedimentos e a renda per capita (29). Explicando, portanto, o maior número absoluto de procedimentos realizado por pessoas cuja região de residência fosse a Sudeste, já que aspectos atrelados ao desenvolvimento econômico e social permitem acesso ampliado a diferentes métodos contraceptivos (24).

No entanto, a relação entre a diferença de regiões urbanas e rurais no que tange às tendências das vasectomias não é bem explícita na literatura. Dois estudos realizados nos Estados Unidos de 2014-2021 (23) e de 2011-2017 (30) encontraram diferenças entre regiões urbanas e rurais, com maior aumento relativo entre aqueles que residiam em regiões rurais (34%) (23), uma menor média de idade dos pacientes e um maior número de filhos (30); enquanto outro estudo, também norte-americano de 2007-2013 (27), não apresentou diferença entre os CVA das regiões. Nesse sentido, a divergência pode ser atribuída aos diferentes períodos de análise, os quais podem estar associados às mudanças legislativas, comportamentais, socioeconômicas e culturais da sociedade estudada, evidenciando as múltiplas variáveis que influenciam no processo de tomada de decisão para escolher o método contraceptivo ideal (24). A urbanização, por sua vez, aparenta exercer dupla influência nas tendências, pois aumenta a oferta de serviços de saúde em áreas urbanas enquanto aumenta as discrepâncias entre regiões urbanas e rurais (24).

Ainda, houve uma parcela importante de dados ignorados na variável raça/cor da pele no período estudado. Apesar da aparente redução na proporção de dados ignorados de 2013 a 2022, não houve preenchimento adequado desta variável no banco de dados em nenhum dos anos analisados segundo a classificação de Romero e Cunha (31). Da mesma maneira que ocorreu neste estudo, um estudo nacional de 2010-2012 (32) e outro de 2009-2018 (33) apontaram 35% e 29% de incompletude nesse sistema de informação, respectivamente. A manutenção, portanto, desse indicador pode estar atrelada ao fato da produção de informação no Brasil ser desorganizada e assistemática, sobretudo no Norte, Nordeste e Centro-Oeste, possivelmente introduzindo vieses nas análises, bem como distorcendo a informação e a realidade (33).

Este estudo não está isento de limitações; as principais limitações estão relacionadas ao seu desenho, que impossibilitou análises em nível individual. Além disso, a indisponibilidade de dados demográficos sobre outras raças/cores da pele impediu a utilização de outras populações. A fonte de dados pode ser uma limitação devido à inserção incorreta dos dados no conjunto de dados e à subnotificação dos procedimentos, especialmente pela quantidade de dados ignorados na variável raça/cor da pele, a qual poderia fornecer informações importantes acerca das condições de vida e saúde das populações ignoradas (32), o que pode ter introduzido equívocos na interpretação dos resultados. Por fim, a análise de países como o Brasil, que possui um grande território e um alto grau de heterogeneidade entre sua população, pode ser uma limitação, uma vez que locais – sobretudo no Norte e Nordeste – de menor porte populacional e com maior dificuldade de acesso à assistência de saúde podem apresentar maior subnotificação (34). No entanto, dividiu-se a análise em regiões, unidades federativas, faixas etárias e raças/cores da pele para minimizar essa limitação.

Apesar das limitações, este estudo é o primeiro a quantificar as tendências das vasectomias no Brasil e em suas regiões, sendo indispensável para a compreensão do cenário da contracepção no Brasil, especialmente após a alteração da lei que regula as esterilizações no Brasil em 2022 (25). Permitindo, assim, que estudos futuros utilizem dos dados apresentados neste manuscrito como objeto de comparação entre dois períodos distintos: antes e depois da alteração da lei. Esta lei altera a idade mínima para solicitar a esterilização no Brasil de 25 para 21 anos (25), o que pode influenciar o processo de tomada de decisão da população, especialmente nas gerações mais jovens. Finalmente, a provisão da VPA, da VPAM e suas tendências são essenciais para avaliar fatores relacionados às mudanças nos padrões de aumento ou diminuição do número de vasectomias.

Em conclusão, as tendências crescentes observadas podem estar atreladas a mudanças nas decisões reprodutivas, eventualmente influenciadas por maior conscientização sobre o planejamento familiar, maior acesso a serviços de saúde especializados, maior confiabilidade no método, além da redução dos estigmas associados à vasectomia. Contudo, disparidades regionais, etárias e raciais ressaltam a importância de políticas públicas direcionadas e estratégias específicas para cada população, visando a promoção da equidade no acesso aos serviços de saúde reprodutiva, bem como o incentivo do planejamento familiar em todo o território brasileiro.

## Data Availability

Os dados estão disponíveis integralmente em https://datasus.saude.gov.br/.
